# Do women prefer a female breast surgeon?

**DOI:** 10.1186/s13584-016-0094-3

**Published:** 2016-12-01

**Authors:** Asnat Groutz, Hadar Amir, Revital Caspi, Eran Sharon, Yifat Amir Levy, Mordechai Shimonov

**Affiliations:** 1 Tel Aviv Sourasky Medical Center, Sackler Faculty of Medicine, Tel Aviv University, Tel Aviv, Israel; 2 The Chaim Sheba Medical Center, Sackler Faculty of Medicine, Tel Aviv University, Ramat Gan, Israel; 3 Rabin Medical Center, Sackler Faculty of Medicine, Tel Aviv University, Petah Tikva, Israel; 4 E. Wolfson Medical Center, Sackler Faculty of Medicine, Tel Aviv University, Holon, Israel; 5 Lis Maternity Hospital, Tel Aviv Sourasky Medical Center, 6 Weizmann Street, Tel Aviv, Israel; 6 Department of Medicine, University of California, La Jolla, San Diego, CA USA

**Keywords:** Breast surgery, Female breast surgeon, Gender preference, Sex preference, Physician-patient relations

## Abstract

**Background:**

Patient preferences regarding the gender of their physicians is a highly sensitive issue, which can be particularly salient in intimate medical situations. Previously published studies found that women tend to prefer female physicians, especially in the case of obstetricians and gynecologists. Data regarding other intimate specialties, such as breast surgery, are scarce. The present study was undertaken to assess gender preferences of women regarding their choice of a breast surgeon.

**Methods:**

Five hundred and fifteen consecutive women who attended breast clinics in two university-affiliated tertiary hospitals were prospectively enrolled. A 25-item anonymous questionnaire was completed by women independently and used to assess their preferences in selecting their breast surgeon. Of the 515 women, 500 (97 % response rate; mean age 50.6 ± 15.4 years) completed the anonymous questionnaire.

**Results:**

Overall, 160 (32 %) women preferred to undergo breast examination by a female breast surgeon, 296 (59 %) had no preference, and only 44 (9 %) preferred a male surgeon. A same-gender preference was significantly and independently associated with younger age of the patients (Odds Ratio = 0.978, 95 % Confidence Interval 0.962–0.994, *P* = 0.007) and being married (Odds Ratio = 0.563, 95 % Confidence Interval 0.347–0.916, *P* = 0.021). However, only small and equal numbers of patients preferred to undergo breast surgery by a female (14 %) or a male (13 %) surgeon, and most patients (73 %) had no gender preferences. Furthermore, the three most important factors, which affected in general the actual selection, were surgical ability (93 %), experience (91.2 %) and knowledge (78.6 %), rather than physician gender per se*.*

**Conclusions:**

Overall, about a third of women prefer a female breast surgeon for their breast examination. Embarrassment during the examination was the major reason for same-gender preference. In contrast, when it comes to breast operations, preference for a female surgeon is less pronounced, with the professional skills of the surgeons becoming the predominant consideration. The fact that almost a third of the potential patients prefer female surgeons with regard to their breast examinations emphasizes the need to increase the number of female surgeons. Such an increase can be achieved through academic and economic changes that will enable more women to specialize in general surgery.

**Trial registration:**

Trial registration is not required for this type of research.

## Background

The significance of gender considerations in choosing a specific physician, in different medical situations, is not well understood. This is particularly important nowadays, when more and more female physicians practice surgical specialties previously dominated by male surgeons, allowing female patients the option to choose a female surgeon. Previous studies have demonstrated women’s preference for female physicians, especially in intimate medical situations such as Obstetrics and Gynecology [1, 2] and Colorectal endoscopy [3–5]. However, to date, only one British study, published in 1998, investigated women’s preferences regarding the gender of their breast surgeon [6]. Results of that study suggested that up to one third of women prefer to be referred to a breast clinic with a female surgeon.

Women made up about 5 % of all physicians in the United States in 1970; the number rose to 24 % in 2001 [7]. Currently medical school enrolment, as estimated by the American Association of Medical Colleges, is about equal between men and women [8]. The number of women entering general surgery has climbed steadily; they now account for more than 35 % of trainees in the United States. Davis et al. [9] found a 22 % relative increase in the percentage of women among medical graduate applicants to general surgery programs between application years 2000 (*N* = 506; 27 %) and 2005 (*N* = 754; 33 %). Additionally, there was a 25 % relative increase in the percentage of women among medical graduates who began general surgery training between academic years 2000–2001 (*N* = 282; 32 %) and 2005–2006 (*N* = 384; 40 %).

In Israel, similar to other Western countries, about half of the medical students (55 %), and of the medical residents (43 %), are women [10]. However, unlike the significant increase in the number of women entering general surgery in the United States, female surgeons in Israel are still a distinct minority, and represent only 20 % of the residents in general surgery programs [11].

The aim of the current study was to explore gender preferences of women regarding their choice of a breast surgeon. Additional relevant characteristics affecting these choices were examined as well.

## Methods

Five hundred and fifteen consecutive female patients, aged 18 and older, who attended breast clinics in two university-affiliated tertiary hospitals, were prospectively enrolled. An anonymous questionnaire was used to assess patient’s preferences in selecting their breast surgeon. The study protocol was reviewed and approved by the E. Wolfson Hospital’s institutional ethical review board which waived the requirement that respondents complete an informed consent form for this study format. Of the 515 women, 500 (97 % response rate; mean age 50.6 ± 15.4 years) completed the anonymous questionnaire. Most patients were referred for either routine breast evaluation (*N* = 217, 43.4 %), previous breast cancer (*N* = 76, 15.2 %), or abnormal mammography (*N* = 54, 10.8 %).

We developed a questionnaire that incorporated items from validated instruments previously used to assess gender preference of women for their obstetricians and gynecologists [1, 2]. The final questionnaire was comprised of 25 items. The first part of the questionnaire included basic socio-demographic information, gender of the patients' regular surgeon, gynecologist and family physician, and previous examination by a male or female breast surgeon. The second part of the questionnaire included questions regarding gender preferences in different medical situations, such as breast examination, or breast surgery. Additionally, patients were asked to identify how specific characteristics of breast surgeons tend to be related to their gender. These included being gentle, sympathetic, tolerant, medical knowledge and surgical skills. The participants answered these questions by circling the word “male”, “female” or “none” next to each characteristic. Each participant was also asked to circle three out of a list of 16 characteristics she considered to be the most important in choosing her breast surgeon. These characteristics included demographic items (age, gender, origin, marital status, parental status, religious status), professional skills (ability, experience, knowledge), qualifications (schools attended, board certification, hospital affiliation, university affiliation), and other qualities (personality, reputation, availability).

Statistical analysis was performed using Student’s *t*-test for continuous data, or Fisher exact test for categorical data. The data are summarized as mean ± SD, or number of responders (percentage) according to the variables. *P* < 0.05 was considered statistically significant. A multivariate binary logistic regression analysis was used to study which variables were independently associated with preference for a female breast surgeon. The statistical analyses were carried out by using the 22th version of the SPSS statistical software (SPSS Inc., Chicago, IL, USA).

## Results

The study population was comprised of 500 consecutive female patients (mean age 50.6 ± 15.4 years). Overall, 141 (28.2 %) patients preferred a female breast surgeon, 54 (10.8 %) preferred a male breast surgeon and 305 (61 %) had no gender preference. Statistical analysis by the various indications for examination failed to reveal any statistically significant differences and therefore all 500 patients were further analyzed as one group.

Analysis by the different indications as well as the demographic and clinical characteristics of the patients are presented in Table 1. Patients who preferred a female breast surgeon were significantly younger than patients who preferred a male surgeon or had no gender preference (46.4 ± 14.2 years versus 52.2 ± 15.5 years, respectively; *P* = 0.0001). Also, the preference for a female breast surgeon was more common among married compared to non-married patients (31.2 % versus 20.3 %, respectively; *P* = 0.016).

**Table 1:** Demographic and clinical characteristics

Mean ± SD, or N (%)	Total participants *N* = 500	Prefer female breast surgeon *N* = 141	Prefer male breast surgeon *N* = 54 No preference *N* = 305	*P*
Age (years)	50.6 ± 15.4	46.4 ± 14.2	52.2 ± 15.5	0.0001
Indications:				NS
Routine examination	217 (46 %)	61 (28.1 %)	156 (71.9 %)	
Previous surgery	76 (16.1 %)	19 (25 %)	57 (75 %)	
Mastitis	11 (2.3 %)	4 (36.4 %)	7 (63.6 %)	
Prior to fertility treatments	17 (3.6 %)	3 (17.6 %)	14 (82.4 %)	
Family history of breast cancer	35 (7.4 %)	14 (40 %)	21 (60 %)	
Pathological biopsy or imaging	54 (11.4 %)	10 (18.5 %)	44 (81.5 %)	
Other indications	62 (13.1 %)	18 (29 %)	44 (71 %)	
Religion:				NS
Jewish	429 (87.7 %)	119 (27.7 %)	310 (72.3 %)	
Christian	24 (4.9 %)	5 (20.8 %)	19 (79.2 %)	
Muslim	25 (5.1 %)	11 (44 %)	14 (56 %)	
Other	11 (2.2 %)	3 (27.3 %)	8 (72.7 %)	
Religious status:				NS
Secular	267 (56.3 %)	65 (24.3 %)	202 (75.7 %)	
Religious	150 (31.6 %)	41 (27.3 %)	109 (72.7 %)	
Extremely religious	57 (12 %)	21 (36.8 %)	36 (63.2 %)	
Marital status:				0.016
Non- married	148 (30 %)	30 (20.3 %)	118 (79.7 %)	
Married	346 (70 %)	108 (31.2 %)	238 (68.8 %)	
Education:				NS
Primary school	41 (8.5 %)	6 (14.6 %)	35 (85.4 %)	
High school	125 (26 %)	36 (28.8 %)	89 (71.2 %)	
College	86 (17.9 %)	31 (36 %)	55 (64 %)	
University	228 (47.5 %)	62 (27.2 %)	166 (72.8 %)	
Gender of the regular surgeon				0.0001
Male	320 (64 %)	60 (18.8 %)	260 (81.3 %)	
Female	110 (22 %)	58 (52.7 %)	52 (47.3 %)	
Gender of the gynecologist				0.009
Male	246 (49.2 %)	56 (22.8 %)	190 (77.2 %)	
Female	215 (43 %)	73 (34 %)	142 (66 %)	
Gender of the family physician				0.025
Male	205 (41 %)	46 (22.4 %)	159 (77.6 %)	
Female	283 (56.6 %)	90 (31.8 %)	193 (68.2 %)	

In Israel, surgeons are the ones that provide breast health services, both preventive and therapeutic. Analysis by previous exposure to female breast surgeons, female gynecologists, or female family doctors is presented in Table 1. One hundred and ten (22 %) patients were previously routinely managed by female surgeons, and 58 (52.7 %) of them preferred a female breast surgeon. Two hundred and fifteen (43 %) patients were routinely managed by female gynecologists, and 73 (34 %) of them preferred a female breast surgeon. Similarly, 283 (57 %) patients were routinely managed by female family physicians, and 90 (31.8 %) of them preferred a female breast surgeon. Those patients who preferred female breast surgeons showed a significantly higher preference for female family physicians rather than a male family physicians or no gender preference (64.8 % versus 18.6 %, respectively; *P* = 0.0001).

Table 2 displays gender preference for non-invasive versus invasive aspects of surgical care. Four aspects were examined: breast examination, general physical examination, breast surgery, and other surgery. There was almost no difference between breast examination and general physical examination: almost a third of all patients preferred to undergo breast examination (32 %), or general physical examination (26 %), by a female surgeon. Embarrassment during the examination was the major reason for same-gender preference. However, in the case of breast surgery, most patients (73 %) had no gender preferences and only a small number of patients preferred a female (14 %) or a male (13 %) surgeon. Interestingly, fewer patients (7 %) preferred to undergo general surgery by a female surgeon, and most (82 %) had no gender preferences.

**Table 2:** Women’s gender preference for breast surgeons by various procedures performed

*N* (%)	Prefer female surgeon	Prefer male surgeon	No preference
Breast examination	160 (32 %)	44 (9 %)	296 (59 %)
General physical examination	131 (26 %)	37 (7 %)	332 (67 %)
Breast surgery	69 (14 %)	65 (13 %)	366 (73 %)
Other surgery	37 (7 %)	55 (11 %)	408 (82 %)

Table 3 shows the features selected by patients as important in choosing a breast surgeon. Of the 16 examined characteristics, the patients ranked surgical ability (93 %), experience (91.2 %) and knowledge (78.6 %) as the top three required qualities of breast surgeons. Other characteristics, such as demographic background, qualifications, and other selected qualities (personality, reputation and availability) were less important.

**Table 3:** Characteristics selected by patients as important in choosing a breast surgeon

Characteristics of surgeons	*N* (%)
Demographics	
Age	52 (10.7 %)
Gender	45 (9.2 %)
Origin	16 (3.3 %)
Marital status	8 (1.6 %)
Parental status	3 (0.6 %)
Religious status	8 (1.6 %)
Professional skills	
Ability (professional)	453 (93 %)
Experience	444 (91.2 %)
Knowledge	383 (78.6 %)
Qualifications	
Board certification	155 (31.8 %)
Schools attended	36 (7.4 %)
Hospital affiliation	129 (26.5 %)
University affiliation	12 (2.5 %)
Other qualities	
Personality	215 (44.1 %)
Reputation	221 (45.4 %)
Availability	198 (40.7 %)

A multiple regression analysis showed that the preference for a female breast surgeon was significantly and independently associated with being younger (Odds Ratio = 0.978, 95 % Confidence Interval 0.962–0.994, *P* = 0.007) and being married (Odds Ratio = 0.563, 95 % Confidence Interval 0.347–0.916, *P* = 0.021).

## Discussion

Patient’s preference regarding the demographic and clinical characteristics of their health care provider may affect health needs, satisfaction and quality of life. In comparison with other possible factors, gender preference is likely to have a stronger impact when choosing health professionals engaged in intimate and psychosocial medical treatment. Indeed, several previously published studies found that women tend to prefer female physicians, especially when it comes to obstetrical/gynecological issues [1, 2, 12, 13]. Data regarding other intimate specialties such as breast surgery are scarce. Results of the present study show that overall about a third of female patients prefer a female breast surgeon. Interestingly, patients exhibited more same-gender preference for breast examination (32 %) than for breast surgery (14 %), or any other surgery (7 %).

Same-gender preference for health care providers was previously reported in up to 35 % of patients [14–16]. Several studies investigated women's preference for the gender of their gynecologist-obstetrician, however, only one previously published study (from almost 20 years ago) investigated patients' gender preference for breast surgeons: In 1997 a female surgeon joined a British specialist breast clinic, which had previously been run by a male surgeon [6]. One hundred consecutive newly referred patients were asked to fill in an anonymous questionnaire regarding their preferences for female or male breast surgeons, and 31 of these preferred a female surgeon. The most striking finding was that none of the women preferred a male surgeon. Patients who preferred a female surgeon were younger than women with no preference. Women who stated a preference for a female surgeon made comments such as “women are easier to talk to” and “I feel less embarrassed with a woman.” Patients who had no preference felt that a surgeon's gender did not affect competence and that the most important issue was to have a good surgeon irrespective of gender.

Results of the present Israeli study, performed 18 years later, are similar to those of the earlier British study: overall, 28.2 % of our patients preferred a female breast surgeon, 61 % had no gender preference, and only 10.8 % preferred a male breast surgeon. Embarrassment during the examination was the major reason for same-gender preference. This finding is also supported by other studies among male and female patients that found embarrassment as one of the major reason for same-gender preference [3, 4].

A multiple regression analysis showed that the preference for a female breast surgeon was significantly and independently associated with younger age of the patients and their marital status. These results are supported by a Greek study that demonstrated correlations between marital status and age to same-gender preference of women for their gynecologist [17]. Results also show that about half of women who were previously exposed to female surgeons preferred female breast surgeons, implying that responder’s experience with female surgeons was positive.

Moreover, the Israeli population is composed of several sub-populations with different demographic, religious and social characteristics that may influence the preference of a same-gender health care provider. We have previously studied gender preferences for obstetricians and gynecologists among communities such as Orthodox Jews [18], Muslim Israeli-Arab women [1], and Israeli Druze women [2] where religiousness and modesty are deeply rooted. All three studies have demonstrated a clear preference for female obstetricians and gynecologists, with the overwhelming reasons given being feeling more comfortable and less embarrassed with females, and the notion that female obstetricians and gynecologists are more gentle during intimate procedures. Similarly, results of the present study demonstrated the same preferences with regard to breast and physical examinations. This implies that women prefer examinations by female surgeons because a) they perceive them to be more gentle/sensitive than male surgeons and b) simply because they are women. Interestingly, although Muslim Israeli-Arab women preferred female obstetricians and gynecologists, personal and professional skills were found to be more important factors when it came to actually making a choice. The current study was not designed to detect statistically significant differences among these ethnic subgroups, however, results show a similar trend for preference for a female breast surgeon among Orthodox Jews (33 %) and Muslim Israeli-Arab women (44 %).

We also investigated gender preference for non-invasive versus invasive surgical procedures. Significantly more patients preferred to undergo breast examination by a female surgeon than a male surgeon (32 % versus 9 %, respectively). Interestingly, there was almost no difference between breast examination (32 %) and general physical examination (26 %), however our study was not powered to detect such a difference. On the other hand, with regard to breast surgery most patients (73 %) had no gender preferences and only a small number of patients preferred a female (14 %) or a male (13 %) surgeon. These findings imply that many women prefer female doctors for intimate physical examination, however, when an invasive surgical procedure with a potential health threat is required, professional skills of the surgeon are more important than his/her gender per se. There are at least two important distinctions between breast examination and breast surgery: the degree of risk and the extent to which the patient is conscious and interacting with the physician, and hence subject to embarrassment. Further studies are required to explore the relative contribution of these factors to the difference in preference for a female surgeon. Moreover, further analysis confirmed that the most important parameters for the responders regarding their overall choice of a breast surgeon were professional skills, such as surgical ability, experience and knowledge. Similarly, other studies among modern and western communities found physician’s professionalism rather than gender as the most important preference factor [19–21].

## Policy implications

In Israel, like other western countries, more than 50 % of Medical School graduates are women. However, the number of female surgeons in Israeli general surgery programs is still relatively low, mainly due to long non-flexible working hours, as well as family and motherhood conflicts. In addition, involvement of female physicians in medical research and their representation in senior clinical and academic positions is still relatively low [11]. Women represent about half of the general population. Previous studies have demonstrated women’s preference for female physicians, especially in intimate medical situations. There is no doubt that patients could more frequently and proactively be given a choice of whether they want to have a female or a male surgeon. Results of the present study show more same-gender preference for breast examination (32 %) than for breast surgery (14 %), or any other surgery (7 %). Embarrassment during the examination was the major reason for same-gender preference. In Israel, surgeons are the ones that provide breast health services, both preventive and therapeutic. The fact that almost a third of the potential patients prefer female surgeons with regard to their breast examinations emphasizes the need to increase the number of female surgeons. Such an increase can be achieved by introducing the idea of a career in surgery to female medical students, and by promoting academic and economic programs to investigate issues influencing the choice of medical specialties among medical school graduates. Moreover, female-friendly training programs, as well as improved working conditions, are needed to encourage more women to practice surgery. These changes require a cooperative, multi-pronged effort on the part of government and academia. Such an effort could include encouraging less working hours and less night shifts, providing financial support necessary for appropriate care of young children and/or providing near hospital child care facilities, providing adequate salaries to senior surgeons in public hospitals, and encouraging the involvement of female physicians in medical research and their representation in senior clinical and academic positions. In addition, an educational campaign could contribute to at least some women being more open to being examined by a male breast surgeon.

## Conclusions

In conclusion, results of the present study show that overall about a third of female patients prefer to undergo breast examination by a breast surgeon of their own gender. Embarrassment during the examination was the major reason for same-gender preference. A same-gender preference was significantly and independently associated with age (being higher in younger patients) and marital status (being higher among married patients).

In comparison with the situation regarding breast examinations, when it comes to breast surgery the percentage of patients who prefer a female surgeon is much lower. It appears that the professional skills of the surgeon are considered to be more important factors than gender per se when invasive surgical procedures are required. In addition, embarrassment is apparently a more salient factor in the examination room, where the patient is consciously interacting with the surgeon, than in the operating theater.

Further and larger studies are required to evaluate other populations in different countries, as gender preferences could vary as a result of cultural differences and differences in health systems.

## Abbreviations

N, number of subjects; NS, non-significant; SD, standard deviation
